# Reluctance to care for patients with HIV or hepatitis B / C in Japan

**DOI:** 10.1186/s12884-016-0822-2

**Published:** 2016-02-05

**Authors:** Koji Wada, Derek R. Smith, Tomohiro Ishimaru

**Affiliations:** Bureau of International Health Cooperation, National Center for Global Health and Medicine, Tokyo, Japan; School of Health Sciences, Faculty of Health and Medicine, University of Newcastle, Ourimbah, Australia; Department of Occupational Health and Safety, Faculty of Public Health, Mahidol University, Bangkok, Thailand; Occupational Health Training Center, University of Occupational and Environmental Health, Kitakyushu, Japan

**Keywords:** Reluctance to care, Hepatitis B/C, HIV, Infection risk, Nurse, Prejudice

## Abstract

**Background:**

Healthcare workers are faced with various professional dilemmas in the workplace, including at times, a reluctance to care for particular patients. This study investigated personal attitudes and factors influencing Japanese nurses’ reluctance to care for patients infected with HIV, Hepatitis B Virus (HBV), or Hepatitis C Virus (HCV).

**Methods:**

Participants completed an anonymous online survey focusing on potential attitudes towards hypothetical patients, awareness of infection risk and their confidence in using precautions to prevent infection. Statistical associations were analyzed using Poisson regression models.

**Results:**

Regarding personal attitudes, 41 % and 18 % of nurses agreed or somewhat agreed that they would be reluctant to care for a hypothetical patient infected with HIV or HBV / HCV, respectively. Reluctance to care for patients with HIV or HBV / HCV was positively associated with prejudicial attitudes and negatively associated with confidence in personal safety precautions. Hypothetical reluctance to care for patients with HBV / HCV was negatively associated with actual previous experience caring for HBV / HCV patients. Older age among nurses (≥50 years) was positively associated with an increased reluctance to care for hypothetical patients with HIV.

**Conclusions:**

Overall, this study suggests that anxiety arising from perceived infection risk and having a prejudicial attitude might affect the acceptance of infected patients, while personal confidence in universal precautions probably mitigates this situation. Improving nurses’ confidence in using universal precautions therefore represents a positive measure that can help reduce prejudice and improve the quality of healthcare services in Japan, as elsewhere.

**Electronic supplementary material:**

The online version of this article (doi:10.1186/s12884-016-0822-2) contains supplementary material, which is available to authorized users.

## Background

Healthcare workers are faced with various professional dilemmas in the workplace, including at times, an unwillingness or reluctance to care for a particular patient [1–3]. These situations most commonly occur when a healthcare worker’s personal beliefs or concerns for their personal safety conflict with the tasks they are expected to provide [4]. Nurses represent one of the largest professional groups in the healthcare workforce, and are well-aware of the risks posed by sharps injuries or body fluid exposure when dealing with patients infected with HIV, Hepatitis B Virus (HBV), or Hepatitis C Virus (HCV) [5, 6]. Effective vaccination is available to protect health care workers [7] from HBV infection, although HCV and HIV still need to be treated if a worker becomes infected [8, 9]. Prevention of needle stick or sharps injuries and body fluid exposure is essential in Japan, as elsewhere, especially for younger health care workers for whom the risk may be greater [10].

Previous studies have shown that some nurses may harbor prejudicial attitudes towards patients infected with certain viruses, particularly as risk behaviors for infection may include sexual transmission or substance abuse [11, 12]. Recent estimates in Japan suggest that the prevalence is 0.71 % for HBV and 0.63 % for HCV, based on blood donor statistics [13]. The community prevalence of HIV is 0.018 % amongst low-risk Japanese men; a rate which is relatively lower than that of many other developed countries [14]. In Japanese hospitals, therefore, it is relatively more common for nurses to care for patients infected with HBV and HCV. HIV positive patients are less commonly encountered in Japanese health care practice, as there are designated hospitals for the care of such patients. As a result, there have been relatively fewer opportunities to address potential prejudice and change nurses’ perceptions of patients infected with HIV in this country.

Stigma and discrimination toward patients infected with blood-borne infectious diseases have been investigated in various studies [15, 16] although health care professionals may underreport their feelings on such matters. It is therefore necessary to address this issue to help improve service delivery and protect health care workers through proper training opportunities [17]. To the best of the authors’ knowledge, few if any studies have been conducted in Japan which investigate reluctance to care for HIV, HBV or HCV patients. This is despite the fact that other Japanese researchers have shown that up to 40 % of non-health care workers might harbor a prejudicial perception of HIV and up to 24 % have shown a prejudicial perception of HBV / HCV [18–20]. The present study aimed, therefore, to determine the factors associated with Japanese nurses’ reluctance to care for patients infected with HIV or HBV / HCV.

## Methods

### Participants and survey method

This study focused on Japanese nurses currently working in hospitals and clinics. To recruit participants, we utilized an internet research company with a database of voluntarily registrants. Of the registrants, 1111 individuals reported currently working as nurses, and were therefore recruited into the study.

#### Questionnaire (independent variables)

The survey tool comprised questions focusing on demographic items such as age and current workplace (either clinics or hospitals), awareness of risk of infection, confidence of precautions for not becoming infected, experience dealing with patients infected with HIV or HBV / HCV, attitude toward patients with HIV or HBV / HCV, and their opinion regarding a hypothetical patient infected with HIV or HBV / HCV (reluctance to care).

The specific item assessing this issue was worded in Japanese, as follows: “If I found out that a patient for whom I must care for was infected with HIV, I would become anxious that I might be infected.” A separate (but similarly worded) statement was used to assess anxiety related to HBV / HCV infection. Regarding their confidence in taking appropriate precautions, the item used was: “I am confident that I could protect myself from infection even when caring for a patient infected with HBV or HCV.” The item assessing prejudicial attitude was worded as: “I would have a biased view that my patient might be a homosexual, have had sexual relations with multiple partners, or be a drug user, if that person was infected with HIV” (with their attitude towards HBV / HCV patients being assessed in a separate item). Responses for these items were measured on a five-point scale (1 = agree; 2 = somewhat agree; 3 = somewhat disagree; 4 = disagree; and, 5 = do not know). To assess any previous experience in dealing with such patients, we asked the following question: “Have you cared for a patient infected with HIV (HBV/HCV in a separate question) during the past 1 year?”, with response options of: 1 = yes, 2 = no, and 3 = do not know or do not want to answer.

#### Questionnaire (outcome)

Nurses’ future behavior towards patients infected with HIV or HBV / HCV were assessed with the item: “I would not want to care for a patient who is infected with HBV / HCV” (HIV patients being assessed in a separate question), with responses again scored on a five-point scale (1 = agree; 2 = somewhat agree; 3 = somewhat disagree; 4 = disagree; 5 = do not know).

#### Statistical analysis

Poisson regression models with robust variance were applied to determine factors associated with a reluctance to care for patients infected with HIV or HBV / HCV among Japanese nurses, since the prevalence of outcome was relatively common [21]. For the analysis, independent variables were reclassified into a three-point scale: 1 = agree; 2 = somewhat agree; 3 = somewhat disagree, disagree and do not know (1 = yes; 2 = no; 3 do not know/do not want to answer). We reclassified the outcome into a two-point scale (1 = agree and somewhat agree, 0 = other), as follows. Firstly, we conducted a univariate analysis with all variables, adjusting for gender and current workplace. Statistical analyses were performed using IBM SPSS Statistics, Version 20.0 (IBM Corp., Armonk, NY, USA).

## Ethics

The study aims and protocol were approved by the institutional ethics committee of the National Center for Global Health and Medicine, Japan. Participants were informed in advance that their participation was voluntary and that no identifying information had been provided to the researchers. Participants who agreed to participate could then access the survey.

## Results

A total of 992 nurses participated in the study, the majority of whom were female and aged between 30 and 49 years (Table 1). Table 2 indicates the distribution of responses for each question relating to HIV or HBV / HCV. A number of nurses reported being anxious about the risk of infection from patients with HIV (agree 13 %; somewhat agree 40 %) or HBV / HCV (agree 15 %; somewhat agree 39 %). Around 50 % of respondents were confident they could protect themselves from infection when caring for patients with HIV (49 %) or HBV / HCV (59 %). There was a large gap between the proportion of nurses who reported previous experience in caring for patients infected with HBV / HCV (67 %) versus those who had previously cared for patients with HIV (11 %). With regard to prejudicial attitudes such as considering that these hypothetical patients might be homosexuals, have multiple sexual partners or be drug users; 39 % of respondents agreed or somewhat agreed for HIV infection and 13 % for HBV / HCV infection. For the item suggesting a reluctance to care, 41 % of nurses agreed or somewhat agreed that they would not want to care for a hypothetical patient infected with HIV and 18 % agreed or somewhat agreed they would not want to care for a patient infected with HBV / HCV.

**Table 1:** Participant demographics (*n* = 992)

	*n*	(%)
Age range		
20–29 years	135	(14)
30–39 years	309	(31)
40–49 years	369	(37)
50+ years	179	(18)
Gender		
Female	879	(89)
Current workplace		
Outpatient clinics	320	(32)
Hospitals	672	(68)

**Table 2:** Distribution of responses by HIV or HBV / HCV status

	HIV		HBV/HCV	
	*n*	(%)	*n*	(%)
Anxiety regarding the potential infection risk from patients with HIV or HBV / HCV				
Agree	133	(13)	145	(15)
Somewhat agree	397	(40)	391	(39)
Somewhat disagree	254	(26)	303	(31)
Disagree	186	(19)	139	(14)
Do not know	22	(2)	14	(1)
Confident that I could protect myself from infection when caring for patients infected with HIV or HBV / HCV				
Agree	125	(13)	179	(18)
Somewhat agree	359	(36)	403	(41)
Somewhat disagree	253	(26)	231	(23)
Disagree	143	(14)	84	(9)
Do not know	112	(11)	95	(10)
Have you cared for a patient infected with HIV or HBV/HCV during the past one year?				
Yes	110	(11)	668	(67)
No	700	(71)	218	(22)
Do not know	182	(19)	106	(11)
Do you believe that a patient infected with HIV or HBV / HCV might be a homosexual, have multiple sexual partners or be a drug user?				
Agree	93	(9)	31	(3)
Somewhat agree	294	(30)	99	(10)
Somewhat disagree	261	(26)	330	(33)
Disagree	304	(31)	512	(52)
Do not know	40	(4)	17	(2)
Would you be reluctant to care for a patient infected with HIV or HBV / HCV?				
Agree	109	(11)	43	(4)
Somewhat agree	294	(30)	136	(14)
Somewhat disagree	266	(27)	325	(33)
Disagree	271	(27)	466	(47)
Do not know	52	(5)	22	(2)

*HBV* hepatitis B virus, *HCV* hepatitis C virus

Table 3 indicates the statistical correlations between reluctance to care for patients infected with HIV or HBV / HCV and other factors. Reluctance to care for patients infected with either HIV or HBV / HCV was positively associated with anxiety regarding the risks of infection (HIV: agree, Odds Ratio (OR) 3.58, 95 % Confidence Interval (95 % CI): 2.75–4.68; somewhat agree OR: 2.92, 95 % CI: 2.26–3.77 and for HBV / HCV: agree OR: 6.36, 95 % CI: 3.96–10.2; somewhat agree OR 2.94, 95 % CI: 1.87–4.63); but negatively associated with being confident of precautions (HIV: agree OR: 0.75, 95 % CI: 0.59–0.95 and for HBV / HCV: agree OR: 0.59, 95 % CI: 0.40–0.88 and somewhat agree OR: 0.64, 95 % CI: 0.49–0.85). Previous experience with infected patients was negatively associated with a reluctance to care for future (hypothetical) HBV / HCV patients only (Yes: OR: 0.75, 95 % CI: 0.58–0.97). Participants over 50 years of age were positively associated with potential unwillingness to care for patients infected with HIV (OR: 1.21, 95 % CI: 1.00–1.48) (Additional file 1: Supporting raw data).

**Table 3:** Statistical correlations with a reluctance to care for a hypothetical patient infected with HIV or HBV / HCV (*n* = 992)

	HIV				HBV / HCV			
	Univariate		Adjusted		Univariate		Adjusted	
	OR	(95 % CI)	OR	(95 % CI)	OR	(95 % CI)	OR	(95 % CI)
Age (y)								
20–29	1.00	-	1.00	-	1.00	-	1.00	-
30–39	1.07	(0.82–1.40)	0.97	(0.78–1.22)	0.62	(0.42–0.91)	0.75	(0.52–1.06)
40–49	1.17	(0.90–1.50)	1.06	(0.86–1.31)	0.68	(0.47–0.97)	0.89	(0.65–1.22)
50+	1.32	(1.03–1.71)	1.21	(1.00–1.48)	0.62	(0.40–0.97)	0.86	(0.59–1.26)
Anxiety regarding the potential infection risk from patients with HIV or HBV / HCV								
Agree	6.51	(5.13–8.25)	3.58	(2.75–4.68)	10.4	(6.78–15.9)	6.36	(3.96–10.2)
Somewhat agree	4.10	(3.21–5.24)	2.92	(2.26–3.77)	4.06	(2.60–6.32)	2.94	(1.87–4.63)
Disagree/somewhat disagree/do not know	1.00	-	1.00	-	1.00	-	1.00	-
Confident that I could protect myself from infection when caring for patients infected with HIV or HBV / HCV								
Agree	0.51	(0.37–0.71)	0.75	(0.59–0.95)	0.42	(0.27–0.65)	0.59	(0.40–0.88)
Somewhat agree	0.81	(0.69–0.95)	1.01	(0.88–1.15)	0.47	(0.34–0.63)	0.64	(0.49–0.85)
Disagree/somewhat disagree/do not know	1.00	-	1.00	-	1.00	-	1.00	-
Have you cared for a patient infected with HIV or HBV/HCV during the past 1 year?								
Yes	1.03	(0.82–1.29)	0.95	(0.80–1.16)	0.76	(0.56–1.00)	0.75	(0.58–0.97)
No	1.00	-	1.00	-	1.00	-	1.00	-
Do not know/do not want to answer	0.75	(0.60–0.95)	0.97	(0.80–1.16)	0.40	(0.21–0.76)	0.59	(0.34–1.03)
Do you believe that a patient infected with HIV or HBV / HCV might be a homosexual, have multiple sexual partners or be a drug user?								
Agree	4.86	(4.09–5.79)	2.77	(2.28–3.36)	6.81	(5.22–8.89)	2.81	(2.03–3.88)
Somewhat agree	3.61	(3.01–4.34)	2.51	(2.08–3.04)	5.06	(3.93–6.51)	3.34	(2.56–4.38)
Disagree/somewhat disagree/do not know	1.00	-	1.00	-	1.00	-	1.00	-

*OR* odds ratio, *CI* confidence interval, *HBV* hepatitis B virus, *HCV* hepatitis C virus

## Discussion

This study examined factors associated with a reluctance to care for hypothetical patients infected with HIV or HBV / HCV among Japanese nurses. As such, it represents one of the first studies of prejudicial attitudes toward patients infected with HIV and HBV / HCV in this country. Our findings suggest that anxiety regarding the perceived risk of infection and having a prejudicial attitude might affect the acceptance of infected patients, while personal confidence in universal precautions probably mitigates this attitude. It is worth noting however, that the prevalence of HIV remains quite low in Japan, and therefore this issue is unlikely to become a higher priority without targeted awareness raising.

Previous research has shown that a certain proportion of healthcare workers are reluctant to care for specific patient groups [22–24] including some early studies of reluctance to care for HIV patients in the US, and elsewhere [25, 26]. In the current study, we found that more nurses reported an unwillingness to care for hypothetical patients infected with HIV (41 %), than for patients infected with HBV / HCV (18 %). In comparison, there are few recent studies which have investigated this issue in developed countries [22], although there have been various investigations undertaken in developing countries or countries with high HIV prevalence rates [23, 24, 27, 28]. It may be that in developed countries, expressing any discriminatory attitude towards infected people is prohibited, as the standard precaution is a fixed protocol for infection control. However, in real life situations, some healthcare workers (regardless of location) may still harbor concerns regarding infected patients. In a study conducted in urban India, for example, 88 % of nurses with a high risk of body fluid exposure and 44 % with a low risk of body fluid exposure reported that they discriminate against people living with HIV in professional situations with a high risk of exposure [29]. Future research is therefore necessary to investigate health care workers’ attitudes towards infected persons in order to develop more effective interventions for reducing of discrimination of this nature.

There is no doubt that nurses may be at risk of HIV or HBV / HCV infection through needlestick injury or fluid exposure during their daily work tasks [5]. However, positive safety climate and confidence in their own skills probably helps improve their acceptance of patients infected with blood-borne infections. In the current study, we found that nurses who had no prior experience caring for patients with HBV / HCV, reported less acceptance of this patient group if on duty. Around two-thirds of respondents described previous experience in caring for patients infected with HBV / HCV, while only 11 % of nurses reported prior clinical experience with HIV-positive patients. However, appropriate training to prevent fluid exposure and needlestick injuries can certainly help minimize negative perceptions and bias against infected patients, as demonstrated by other studies [19, 30, 31].

Negative attitudes towards patients infected with HBV and HCV also negatively affected nurses’ perception of their duty, with 13 % reporting that they would be reluctant to care for patients with HBV / HCV. The most likely route of HCV infection is by large or repeated percutaneous exposure to infected blood, such as during transfusion or injecting drug use [32]. In Japan, HCV transmission by fibrinogen concentrate historically represented one of the major sources of infection [33]. However, in Japanese healthcare settings, nurses may also encounter patients who have been infected with HCV and HIV due to drug use [12]. Caring for drug users (especially IV drug users), poses a challenging moral dilemma in Japan, meaning that some nurses may be reluctant to care for these patients when on duty [34]. A similar phenomenon might also apply for homosexual men, as the majority of Japanese HIV infections are reported to be among this demographic [35]. At a broader level, the acceptance of sexual minorities in Japan is still in its early stages [36, 37], and some nurses may therefore experience difficulty in communicating with homosexual people and being confronted with the issues faced by HIV infected patients [38].

It is important to note that older Japanese nurses have probably lived through a more severe era of HIV panic, when compared to their younger counterparts. Until the late 1990s when antiretroviral therapy was developed, for example, HIV was recognized as a progressive illness with an invariably fatal outcome. As a result, many Japanese nurses were particularly afraid of HIV infection via needlestick injury or fluid exposure [39]. In addition, previous research suggests that older people may harbor greater prejudice toward others living with HIV [18, 40]; a finding consistent with our current study. Japanese citizens were also affected by the so-called “AIDS panic” characterized by highly sensational media reports in 1986 and 1987, after the first and second cases of Japanese HIV infection were identified [41, 42]. Since that time, however, improvements in antiretroviral therapy have changed HIV infection more towards a chronic, manageable disease; while the relatively low prevalence of the disease in Japan means that younger nurses tend to have less discriminatory attitudes [40]. Even so, this situation clearly highlights the need for healthcare workers in managerial positions (who are usually senior) to implement appropriate educational opportunities aimed at eliminating discrimination toward patients infected with HIV and other infectious diseases.

There are certain limitations that may have affected the current study, namely that it was cross-sectional in design and therefore, causal relationships could not be inferred. In addition, as the study was conducted as an internet based survey, the results may not be generalizable to all nurses in Japan. However, as participation was online and anonymous, concerns regarding their free expression of bias should have been minimized. A further limitation is that we only asked the nurses’ perception towards hypothetical patients with HIV, HBV, or HCV, rather than their actual experience of refusing to care for them in clinical practice. Additionally, the questionnaires for reluctance care and other variables were not validated, as there is no existing tool currently available in Japanese. This, in itself, represents an ideal opportunity for future research in the field. It is also worth noting that our survey tool only obtained limited demographic information on the participants. Additional studies should take these points into account.

## Conclusion

In conclusion, this study suggests that some Japanese nurses with a reluctance to care for HIV or HBV / HCV infected patients may be aware of the risk of infection but lack confidence in taking precautions against these diseases. Hospitals should therefore provide increased educational opportunities for nurses to improve their confidence in universal precautions and help mitigate prejudicial attitudes toward infected patients to improve accessibility and quality of services for this patient group in Japan, as elsewhere.

## Additional file

Additional file 1:
**Supporting raw data.** (XLSX 50 kb)

## Abbreviations

95 % CI: 95 % confidence interval
HBV: Hepatitis B Virus
HCV: Hepatitis C Virus
OR: odds ratio

## References

[1] Combs EW (1996). Home health, AIDS, and refusal to care. Home Healthc Nurse.

[2] Huerta SR, Oddi LF (1992). Refusal to care for patients with human immunodeficiency virus/acquired immunodeficiency syndrome: issues and responses. J Prof Nurs.

[3] Nuttall CK (2007). Conscientious objection: justified or just refusal to care?. J Perioper Pract.

[4] Cady RF (2007). Refusal to care. JONAS Healthc Law Ethics Regul.

[5] Wilburn SQ, Eijkemans G (2004). Preventing needlestick injuries among healthcare workers: a WHO-ICN collaboration. Int J Occup Environ Health.

[6] Mitsui T, Iwano K, Masuko K, Yamazaki C, Okamoto H, Tsuda F (1992). Hepatitis C virus infection in medical personnel after needlestick accident. Hepatology.

[7] Holmberg S, Suryaprasad A, Ward J (2012). Updated CDC recommendations for the management of hepatitis B virus-infected health-care providers and students. MMWR Recomm Rep.

[8] Chou R, Hartung D, Rahman B, Wasson N, Cottrell E, Fu R (2013). Comparative effectiveness of antiviral treatment for hepatitis C virus infection in adults: a systematic review. Ann Intern Med.

[9] Maartens G, Celum C, Lewin S (2014). HIV infection: epidemiology, pathogenesis, treatment, and prevention. Lancet.

[10] Yoshikawa T, Wada K, Lee JJ, Mitsuda T, Kidouchi K, Kurosu H (2013). Incidence rate of needlestick and sharps injuries in 67 Japanese hospitals: a National Surveillance Study. PLoS ONE.

[11] Hamama L, Tartakovsky E, Eroshina K, Patrakov E, Golubkova A, Bogushevich J, et al. Nurses’ job satisfaction and attitudes towards people living with HIV/AIDS in Russia. Int Nurs Rev. 2014;61(1):131–9. 10.1111/inr.12074 24308493

[12] Wada K, Greberman SB, Konuma K, Hirai S (1999). HIV and HCV infection among drug users in Japan. Addiction.

[13] Tanaka J, Koyama T, Mizui M, Uchida S, Katayama K, Matsuo J, et al. Total numbers of undiagnosed carriers of hepatitis C and B viruses in Japan estimated by age- and area-specific prevalence on the national scale. Intervirology. 2011;54(4):185–95. 10.1159/000324525 21454956

[14] Gilmour S, Li J, Shibuya K (2012). Projecting HIV Transmission in Japan. PLoS ONE.

[15] Nyblade L, Stangl A, Weiss E, Ashburn K (2009). Combating HIV stigma in health care settings: what works?. J Int AIDS Soc.

[16] Singh D, Chaudoir SR, Escobar MC, Kalichman S (2011). Stigma, burden, social support, and willingness to care among caregivers of PLWHA in home-based care in South Africa. AIDS Care.

[17] Stewart KE, DiClemente RJ, Ross D (1999). Adolescents and HIV: theory-based approaches to education of nurses. J Adv Nurs.

[18] Eguchi H, Wada K, Smith DR (2014). Sociodemographic factors and prejudice toward HIV and hepatitis B/C status in a working-age population: results from a national, cross-sectional study in Japan. PLoS ONE.

[19] Eguchi H, Wada K (2013). Knowledge of HBV and HCV and individuals’ attitudes toward HBV- and HCV-infected colleagues: a national cross-sectional study among a working population in Japan. PLoS ONE.

[20] Sasaki N, Wada K, Smith DR, Wang G, Ohta H, Shibuya A (2014). Hepatitis screening in Japanese individuals of working age and prejudice against infected persons in the workplace. J Occup Health.

[21] Coutinho L, Scazufca M, Menezes PR (2008). Methods for estimating prevalence ratios in cross-sectional studies. Rev Saude Publica.

[22] Richmond JA, Dunning TL, Desmond PV (2007). Health professionals’ attitudes toward caring for people with hepatitis C. J Viral Hepat.

[23] Andrewin A, Chien LY (2008). Stigmatization of patients with HIV/AIDS among doctors and nurses in Belize. AIDS Patient Care STDS.

[24] Sekoni OO, Owoaje ET (2013). HIV/AIDS stigma among primary health care workers in Ilorin Nigeria. Afr J Med Med Sci.

[25] Lewis CE, Montgomery K (1992). Primary care physicians’ refusal to care for patients infected with the human immunodeficiency virus. West J Med.

[26] Gillon R (1987). Refusal to treat AIDS and HIV positive patients. Br Med J (Clin Res Ed).

[27] Manganye BS, Maluleke TX, Lebese RT (2013). Professional nurses’ views regarding stigma and discrimination in the care of HIV and AIDS patients in rural hospitals of the Limpopo province South Africa. Afr J AIDS Res.

[28] Ahsan Ullah AK (2011). HIV/AIDS-related stigma and discrimination: a study of health care providers in Bangladesh. J Int Assoc Physicians AIDS Care.

[29] Ekstrand ML, Ramakrishna J, Bharat S, Heylen E (2013). Prevalence and drivers of HIV stigma among health providers in urban India: implications for interventions. J Int AIDS Soc.

[30] Wang G, Wada K, Hoshi K, Sasaki N, Ezoe S, Satoh T (2013). Association of knowledge of HIV and other factors with individuals’ attitudes toward HIV infection: a national cross-sectional survey among the Japanese non-medical working population. PLoS ONE.

[31] Sengupta S, Banks B, Jonas D, Miles M, Smith G (2011). HIV interventions to reduce HIV/AIDS stigma: a systematic review. AIDS Behav.

[32] Alter M (2007). Epidemiology of hepatitis C virus infection. World J Gastroenterol.

[33] Yasunaga H (2007). Risk of authoritarianism: fibrinogen-transmitted hepatitis C in Japan. Lancet.

[34] Wada K (2011). The history and current state of drug abuse in Japan. Ann N Y Acad Sci.

[35] National Institute of Infectious Diseases. HIV/AIDS in Japan, 2013. HIV/AIDS in Japan, 2013. http://www.nih.go.jp/niid/en/basic-science/865-iasr/5000-tpc415.html.Accessed on 20th July 2015.

[36] Lunsing W (2005). LGBT rights in Japan. A Journal of Social Justice.

[37] McLelland M, Suganuma K (2009). Sexual minorities and human rights in Japan: an historical perspective. The International Journal of Human Rights.

[38] Röndahl G, Innala S, Carlsson M (2006). Heterosexual assumptions in verbal and non‐verbal communication in nursing. J Adv Nurs.

[39] Glad J, Tan WN, Erlen JA (1995). Fear of AIDS, homophobia, and occupational risk for HIV. A staff development challenge. J Nurs Staff Dev.

[40] Li L, Liang LJ, Lin C, Wu Z, Wen Y (2009). Individual attitudes and perceived social norms: Reports on HIV/AIDS-related stigma among service providers in China. Int J Psychol.

[41] Ikegami C (1997). HIV prevention and community-based organizations in Japan. J Acquir Immune Defic Syndr Hum Retrovirol.

[42] Hirata S, Watanabe M, Katsuno S (1995). AIDS in the Japanse Mass Media: Content Analysis of Articles about AIDS from 1982 to 1992 in the Japanese Newspaper. Jpn J Health Hum Ecol.

